# Supplementary material for: Free choice shapes normalized value signals in medial orbitofrontal cortex
Source: Nat Commun. 2018 Jan 11;9:162. doi: 10.1038/s41467-017-02614-w (PMC5764979; doi:10.1038/s41467-017-02614-w)
Supplement: Supplementary file 1 — Supplementary Information [file 41467_2017_2614_MOESM1_ESM.pdf]

Supplementary Figures

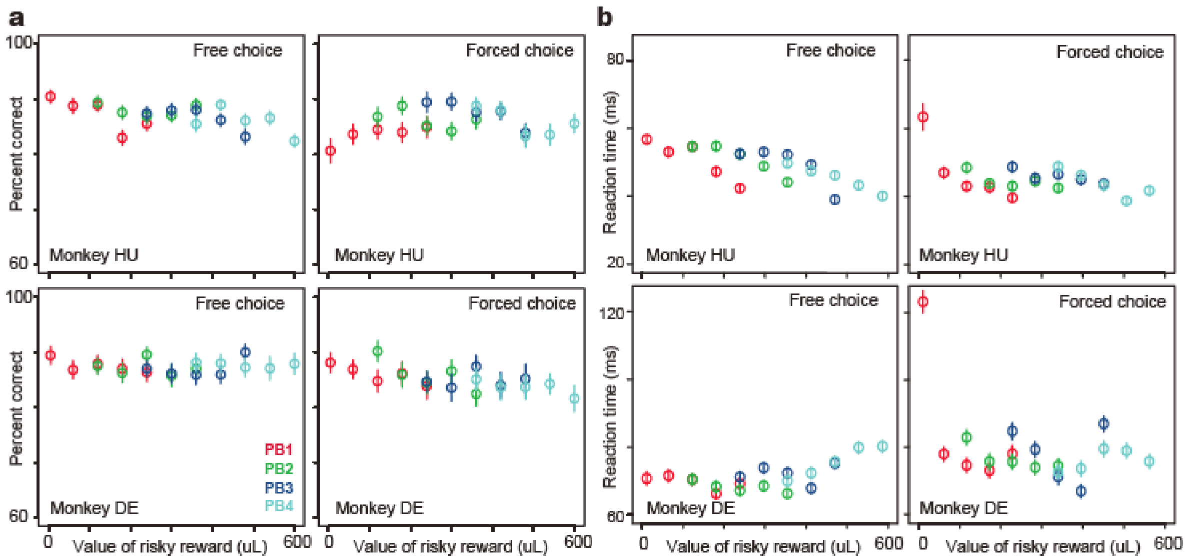

Supplementary figure 1. Effect of the expected values of risky and safe options on behavioral measures of monkeys.

(**a**) Plots of the percent correct trials of two monkeys in twenty lottery pairs during free and forced choice trials. Mean and s.e.m. are plotted. Statistical differences in each of free and forced choice trials were tested by using One-way ANOVA (Monkey HU: Free choice, n = 999, df = 19, P < 0.001, F = 3.17; Forced choice, n= 1011, df = 19, P = 0.006, F = 2.02; Monkey DE: Free choice, n = 970, df = 19, P = 0.95, F = 0.54; Forced choice, n= 967, df = 19, P = 0.60, F = 0.89). Statistical differences between free and forced choice trials were tested by using Two-way ANOVA (Monkey HU: n = 2010, df = 1, P = 0.20, F = 1.62; Monkey DE: n = 1927, df = 1, P = 0.008, F = 7.00). (**b**) Plots of the saccadic reaction times of two monkeys in twenty lottery pairs during free and forced choice trials. Mean and s.e.m. are plotted. Statistical differences in each of free and forced choice trials were tested by using One-way ANOVA (Monkey HU: Free choice, n = 9979, df = 19, P < 0.001, F = 15.3; Forced choice, n= 6036, df = 19, P < 0.001, F = 8.60; Monkey DE: Free choice, n = 9604, df = 19, P < 0.001, F = 5.31; Forced choice, n = 5787, df = 19, P < 0.001, F = 24.4). Statistical differences between free and forced choice trials were tested by using Two-way ANOVA (Monkey HU: n = 16015, df = 1, P < 0.001, F = 19.5; Monkey DE: n = 15391, df = 1, P < 0.001, F = 137.5). Note that mean reaction time was short because monkeys can predict the onset time of the visual signals to move eyes.

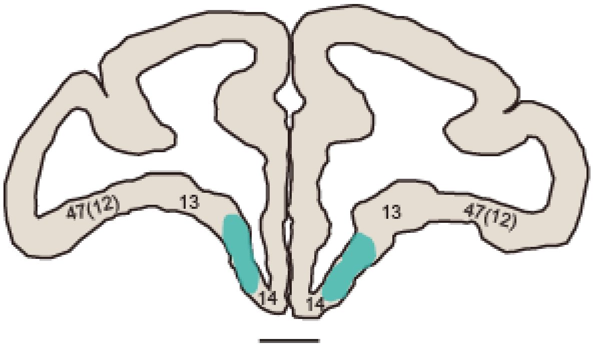

Supplementary figure 2. Recording area of mOFC neurons.

This drawing of recording area (blue areas) was based on a sagittal MR image. Neurons were recorded mostly from medial part of OFC in area 14 (14O, orbital part of area 14) at A32-A34 anterior-posterior (A-P) level, but not at ventromedial wall of area 14 (14M, medial part of area 14). The recorded area was identified according to the atlas in Paxinos Rhesus Monkey (http://scalablebrainatlas.incf.org/main/index.php). Black scale bar indicated 5 mm.

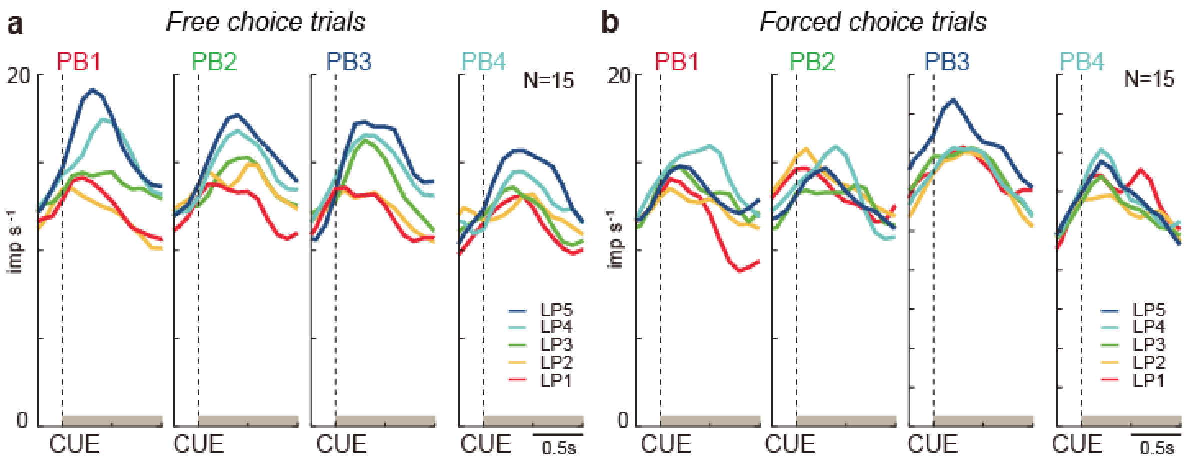

Supplementary figure 3. Relative value signals during free and forced choice trials.
(**a**) Activity histogram of 15 mOFC neurons modulated by relative values of options during cue period (EVr+EVs- type). Activity in free choice trials was represented for twenty lottery pairs. The figure is same as Fig. 2c in the main text. (**b**) Activity histogram of the same 15 mOFC neurons in a, but for the activity during forced choice trials.

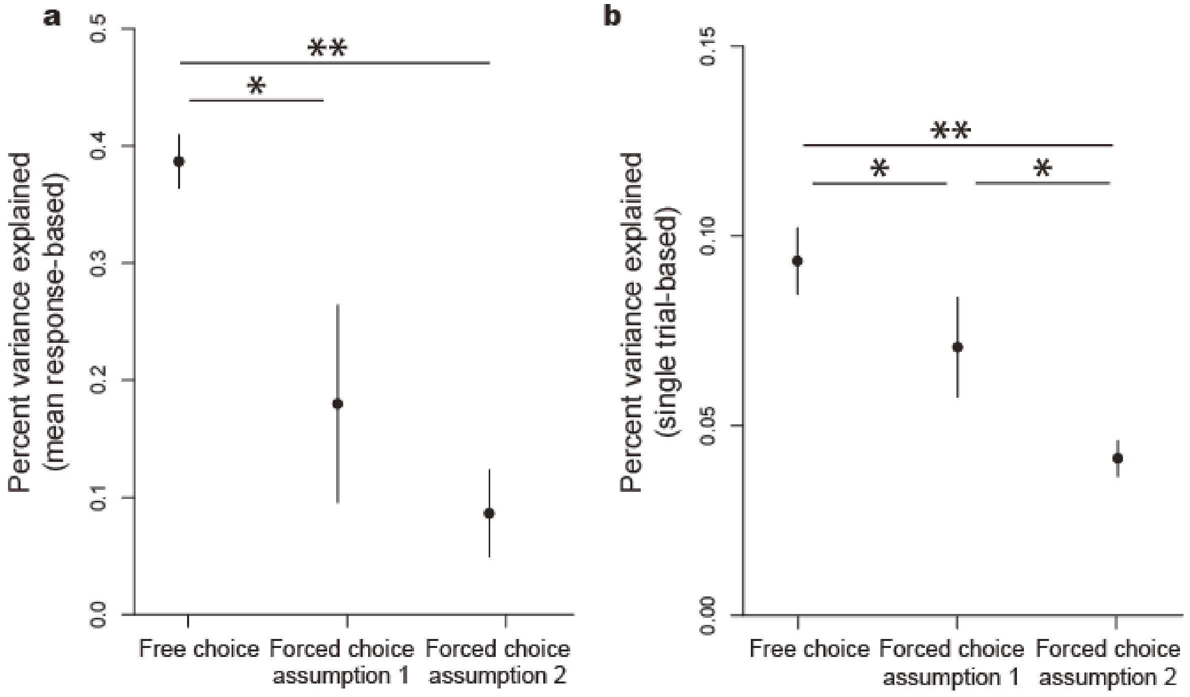

Supplementary figure 4. Percentage of variance explained by the advanced fractional model.

(**a**-**b**) Plot of the percentage of the variance explained by the advanced fractional model during free choice trials, during forced choice trials under assumption 1, and during forced choice trials under assumption 2. The values were estimated based on the mean response data (**a**) and the single trial-based data (**b**), respectively. Single and double asterisks indicate statistical significance level at $P < 0.05$ and $P < 0.01$, respectively, by using paired t-test (Mean response-based; n = 81 and df = 80 for each: Free vs. Forced assumption 1, P = 0.002, t = 3.18; Free vs. Forced assumption 2, $P < 0.001$, t = 5.28; Forced assumption 1 vs. Forced assumption 2, P = 0.22, t = 1.25; Single trial-based; n = 81 and df = 80 for each: Free vs. Forced assumption 1, P = 0.042, t = 2.07; Free vs. Forced assumption 2, P < 0.001, t = 6.49; Forced assumption 1 vs. Forced assumption 2, P = 0.029, t = 2.23). See methods for detail of the assumptions. Mean and s.e.m. are plotted.

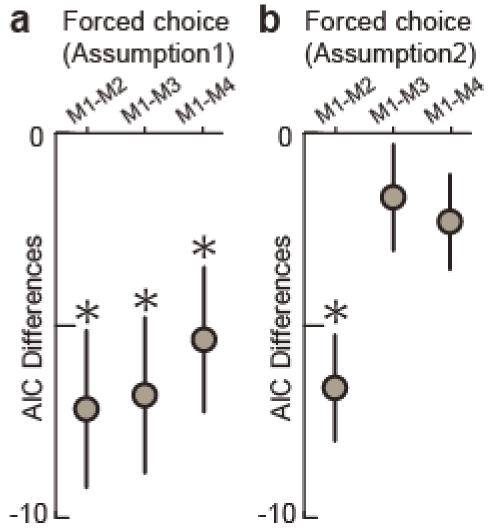

Supplementary figure 5. AIC differences between models in the forced choice trials.
(**a**) Plot of the AIC differences between model 1 and the other three models were shown during forced choice trials under assumption 1. (**b**) Same as A, but for the AIC differences during forced choice trials under assumption 2. Asterisk indicates statistical significance of the differences from zero at $P < 0.01$ using one sample t-test (Forced choice assumption 1: $n = 81$ and $df = 80$ for each, M1-M2, $P < 0.001$, $t = -3.51$; M1-M3, $P = 0.001$, $t = -3.36$; M1-M4, $P = 0.005$, $t = -2.86$) (Forced choice assumption 2: $n = 81$ and $df = 80$ for each, M1-M2, $P < 0.001$, $t = -4.81$; M1-M3, $P = 0.230$, $t = -1.21$; M1-M4, $P = 0.066$, $t = -1.87$). Mean and s.e.m. are plotted. Note that model performance in the forced choice trials were better under the assumption 1 compared to the assumption 2 (Supplementary Fig. 4).

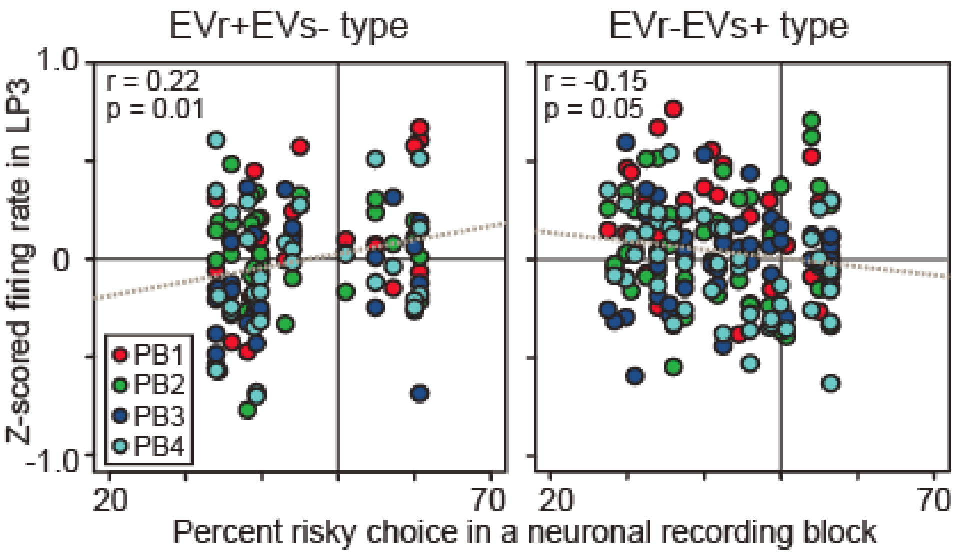

Supplementary figure 6. Divisively normalized value signals were correlated with monkey's risk-attitude.

Plots of the z-scored firing rates observed in the 81 activity of mOFC relative value coding neurons against the percentage of the risky choice during a block of neuronal recordings. Dashed gray lines indicate regression slops. Correlation coefficients and statistical significance were shown. Firing rates of each relative value coding neurons were standardized in each payoff block 1 to 4, respectively.

Supplementary Table

Supplementary Table 1. Correlation coefficients among relative expected values and other possible simple explanatory variables.

	EVr	EVs	EV chosen	Risky choice
Fractional value	0.58	0.18	0.38	0.56
Value difference	0.53	0.00	0.29	0.68
Range normalized value	0.80	0.41	0.63	0.59

EVr: expected values of risky options, EVs: expected values of safe options, EV chosen: expected values of chosen options, Risky choice: choice of risky options defined as a dummy variable indicating whether or not the risky option was chosen. Fractional value: EVr/(EVr + EVs), Value difference: EVr - EVs, Range normalized value: (EVr - Vmin)/(Vmax - Vmin). See methods for the definition of the relative value models.
